# Cofilin Inhibition Ameliorates PIEZO2 and AMPA Dysfunction in a Mouse Model of Angelman Syndrome

**DOI:** 10.1523/JNEUROSCI.0965-25.2025

**Published:** 2025-10-09

**Authors:** Luis O. Romero, Manisha Bade, Elisa Carrillo, Sonia Paz-López, Syed A. M. Hasan, William James Antonisamy, Vasanthi Jayaraman, Zahoor A. Shah, Valeria Vásquez, Julio F. Cordero-Morales

**Affiliations:** ^1^Department of Biochemistry and Molecular Biology, Center for Membrane Biology, University of Texas Health Science Center at Houston, Houston, Texas 77030; ^2^The University of Texas MD Anderson Cancer Center, UTHealth Houston Graduate School of Biomedical Sciences, The University of Texas Health Science Center at Houston, Houston, Texas 77030; ^3^Department of Medicinal and Biological Chemistry, College of Pharmacy and Pharmaceutical Sciences, The University of Toledo, Toledo, Ohio 43614

**Keywords:** AMPA receptor, Angelman syndrome, cofilin, hippocampal neurons, PIEZO2, sensory neurons

## Abstract

Angelman syndrome (AS) is a neurogenetic disorder characterized by motor coordination and cognitive deficits. In AS, hippocampal neurons show reduced filamentous (F-)actin, a decrease we also reported in dorsal root ganglia (DRG) neurons, along with impaired mechanosensitive ion channel activity. Currently, there are no pharmacological targets to prevent the decrease of F-actin in AS. Here, we utilize a first-in-class selective cofilin inhibitor (SZ-3) to restore PIEZO2 function in DRG neurons and glutamate-evoked currents in hippocampal neurons from AS mice. Using atomic force microscopy, we demonstrate that inhibiting cofilin, an actin-severing protein, with SZ-3 increases cellular stiffness by stabilizing the actin cytoskeleton. Furthermore, systemic administration of SZ-3 in male and female AS mice enhances their performance in the rotarod and T-maze tests. These findings support that cytoskeletal dysregulation contributes to impaired ion channel function and behavioral deficits and that actin-binding proteins could serve as a target for enhancing motor coordination and spatial learning in AS.

## Significance Statement

Angelman syndrome (AS) is a severe neurogenetic disorder characterized by significant motor and cognitive impairments; however, effective treatments remain elusive. Recent evidence implicates deficits in the mechanosensitive PIEZO2 channel and α-amino-3-hydroxy-5-methyl-4-isoxazole propionic acid (AMPA) receptor function, as well as cytoskeletal abnormalities in AS pathology. Our study identifies cofilin, an actin-binding protein, as a regulator of ion channel function. We demonstrate that pharmacological inhibition of cofilin restores PIEZO2 channel and AMPA receptor activities, enhances neuronal excitability, and improves motor coordination and learning in a mouse model of AS. These findings reveal a novel mechanism by which actin dynamics influence sensory and cognitive function.

## Introduction

UBE3A is an E3 ubiquitin ligase that transfers a ubiquitin molecule from E2 proteins to substrates, tagging them for proteasomal degradation (Scheffner et al., 1993; Lopez et al., 2018). Loss-of-function (LOF) mutations or the loss of *UBE3A* expression result in Angelman syndrome (AS; Knoll et al., 1989; Kishino et al., 1997; Clayton-Smith and Laan, 2003). AS is a neurogenetic disorder featuring cognitive dysfunction, impaired motor coordination, balance deficits, gastrointestinal dysfunction, and seizures (Tan et al., 2011; Larson et al., 2015; Buiting et al., 2016; Wheeler et al., 2017; Duis et al., 2022). *UBE3A* is genetically imprinted (i.e., a process that leads to monoallelic gene expression) in most neurons of the central nervous system (CNS) and in large-diameter mechanoreceptor neurons of the dorsal root ganglia (DRG; Lopez et al., 2017; McCoy et al., 2017; Tucci et al., 2019). Thus, loss of the maternal allele yields UBE3A-deficient neurons, leading to AS (Buiting et al., 2016).

Proprioceptive behaviors are among the affected sensory processing patterns observed in individuals with AS (Walz and Baranek, 2006). PIEZO2 is a mechanosensitive ion channel expressed in DRG neurons that innervate the skin and muscle spindles, where it mediates touch and proprioception (Coste et al., 2010; Ranade et al., 2014; Woo et al., 2014, 2015; Chesler et al., 2016; Szczot et al., 2021; Nagel and Chesler, 2022). Humans with *PIEZO2* LOF and individuals with AS exhibit similar proprioceptive deficits, including impaired balance and unsteady gait (Larson et al., 2015; Chesler et al., 2016; Wheeler et al., 2017). Recently, we proposed a model in which the loss of *UBE3A* expression, in DRG neurons from an AS mouse model, increases cofilin, decreases filamentous (F-)actin, and consequently downregulates PIEZO2 membrane expression and function (Romero et al., 2023). Importantly, PIEZO2 activity is modulated by the cytoskeleton (Romero et al., 2020; Wang et al., 2022). Hence, interventions aimed at stabilizing F-actin may enhance PIEZO2 activity and improve neuronal function.

Individuals with AS and the corresponding mouse models show impaired synaptic plasticity, which is thought to contribute to the cognitive dysfunction underlying their abnormal spatial and context-dependent learning (Jiang et al., 1998; Miura et al., 2002; Fink et al., 2017). The α-amino-3-hydroxy-5-methyl-4-isoxazole propionic acid (AMPA) receptors are glutamate-gated cation channels that mediate fast excitatory transmission in the CNS (Henley and Wilkinson, 2013, 2016; Diering and Huganir, 2018), and a decrease in their activity has been associated with cognitive decline (Chang et al., 2006; Henley and Wilkinson, 2013; Ding et al., 2016). Moreover, the loss of *Ube3a* in neurons results in decreased activity of AMPA receptors (Greer et al., 2010). Taken together, dysregulation of AMPA receptors may contribute to the cognitive deficits observed in AS, underscoring the potential for novel strategies aimed at increasing AMPA receptor activity.

Actin regulates membrane protein trafficking (Cingolani and Goda, 2008; Zech et al., 2012; Sasaki et al., 2014; Aiken and Holzbaur, 2021). Indeed, disrupting F-actin with latrunculin A reduces PIEZO2 membrane expression and function (Romero et al., 2023). Cofilin is an actin-binding protein that promotes the disassembly of F-actin by binding to and severing it into shorter fragments (Bamburg, 1999; Maciver and Hussey, 2002; Huang et al., 2006; Wioland et al., 2017). We recently developed the first-in-class selective cofilin inhibitor (SZ-3) that reduces cofilin-mediated F-actin depolymerization both in vitro and in vivo (Alaqel et al., 2022; Bahader et al., 2023; Almarghalani et al., 2024). SZ-3 treatment improved neurological and cognitive functions following intracerebral hemorrhage and traumatic brain injury in mice (Bahader et al., 2023; Almarghalani et al., 2024). Moreover, we have shown that SZ-3 can protect microglial and neuroblastoma cells from thrombin-induced apoptosis in vitro (Alaqel et al., 2022). Although the loss of the maternally inherited *UBE3A* gene disrupts several proteins, our study focuses specifically on PIEZO2 and AMPA receptors. We identify that cofilin activity regulates both channels in the context of AS. Pharmacological inhibition of cofilin with SZ-3 enhances neuronal function and improves motor and cognitive performance.

## Material and methods

### Animals

Mice procedures described below were reviewed and approved by the University of Texas Health Science Center at Houston (UTHealth) Animal Welfare Committee (UTHealth AWC protocol number, AWC-23-0093) and the Center for Laboratory Animal Medicine and Care and by the Institutional Animal Care and Utilization Committee at the University of Toledo Health Science Campus (Ethics Reference Number 400095-01-UT). All methods were carried out according to approved guidelines. Adult (2- to 6-month-old) mice were housed with a 12 h light/dark cycle at 21°C with 40–60% humidity and with food and water *ad libitum*. We bred mice from our in-house colony, and their genotype was confirmed using previously published protocols (Jiang et al., 1998; Judson et al., 2016). We used a two-step breeding scheme. Briefly, we first crossed female WT with male *Ube3a^m+/p−^* to generate heterozygous offspring, in which half of the progeny were *Ube3a^m+/p−^*, with no AS phenotype. *Ube3a^m+/p−^* heterozygous females were crossed with WT males to generate *Ube3a^m−/p+^* mice, in which half of the progeny constitute AS mice displaying the corresponding phenotype. After confirming the genotype, the progenies were used for experiments. We also used the WT mouse strain (C57BL/6J; Stock Number 000664) and *Ube3a^m–/p+^* mice obtained from The Jackson Laboratory, strain C57BL/6 *Ube3a^tm1Alb^* (B6 AS; Stock Number 016590).

### Mouse DRG neurons

Primary cultures of mouse DRG neurons were obtained from 8–12-week-old male *Ube3a^m–/p+^* (maternal transmission) mice. Mice were anesthetized with isoflurane and killed by cervical dislocation. DRGs were dissected and kept on ice in 1× Hank’s balanced salt solution (HBSS without CaCl_2_ and MgCl_2_). Next, DRGs were incubated with 1 mg/ml collagenase B (Sigma-Aldrich) in HBSS at 37°C and 5% CO_2_ and then, after 1 h, were dissociated in a medium without serum. The cell suspension solution was centrifuged for 8 min at 62 × *g*. The pellet was resuspended in Dulbecco’s modified Eagle medium (DMEM; Invitrogen) complete media containing 1% penicillin–streptomycin (Invitrogen), 1% MEM vitamin solution (Invitrogen), 1% l-glutamine (Invitrogen), and 10% horse serum (Invitrogen). Cells were plated on poly-l-lysine (Sigma-Aldrich)-treated glass coverslips in 24-well plates. All mouse DRG neurons were kept at 37°C, with 95% relative humidity and 5% CO_2._ Cells were used in experiments after 24–48 h.

### siRNA-mediated knockdown

Primary cultures of mouse DRG neurons were transfected with Lipofectamine RNAiMAX Transfection Reagent (Thermo Fisher Scientific), according to the manufacturer’s protocols. The siRNA concentration was 60 nM for *Cofilin* (AA959946, Horizon Discovery Ltd.) or the silencer negative control. The transfections were done with antibiotic-free media. After 6 h of transfection, the medium was replaced with fresh media containing antibiotics. All cells were used 48 h after transfection. For electrophysiology experiments, cells were also cotransfected with siGLO Green Transfection Indicator (Dharmacon).

### Cell culture

Human Merkel cell carcinoma cell line (MCC13; Cell Bank Australia reference number, CBA1338) was obtained from Sigma-Aldrich and cultured according to the manufacturer’s protocol. MCC13 cells were cultured in RPMI1640 (with 2 mM l-glutamine and 25 mM HEPES; Sigma-Aldrich), 5% penicillin–streptomycin, and 10% FBS. Human embryonic kidney cells (HEK293) obtained from the American Type Culture Collection were cultured in DMEM, supplemented with 10% fetal bovine serum and 1% penicillin–streptomycin. All cultured cells were maintained at 37°C, with 95% relative humidity and 5% CO_2_. Cells were incubated in media supplemented with 10 µM SZ-3 for 18 h before G-/F-actin quantification, atomic force microscopy (AFM), and electrophysiology experiments.

### Cell transfections

For electrophysiology, we used Lipofectamine 2000 (Thermo Fisher Scientific) according to the manufacturer’s instructions. MCC13 cells were grown in six-well plates to 90% confluency and cotransfected with 1.2 µg/ml of Z-lock cofilin (Addgene #141137) and 50 ng/ml GFP-pMO (a pcDNA3.1-based vector with the 5′ and 3′ untranslated regions of the beta-globin gene). HEK 293 cells were grown in six-well plates to 70% confluency and cotransfected with 1 µg/ml AMPA and NMDA receptors, 50 ng/ml rTRPV1-pMO, 500 ng/ml hTRPA1-pMO, or 1 µg/ml rASIC1and 20–50 ng/ml GFP-pMO.

### Protein expression determination

The cytoskeletal fraction of SZ-3- (10 µM, 18 h) and DMSO-treated MCC13 cells was extracted with the globular (G-)actin/ F-actin in vivo assay kit (Cytoskeleton) to determine the G- and F-actin forms according to the manufacturer's instructions. The two fractions of each sample were loaded in equivalent volumes in Mini-PROTEAN TGX Stain-free Precast Gels (Bio-Rad Laboratories) and dry transferred to an Immun-Blot PVDF Membrane (Bio-Rad Laboratories). Mouse monoclonal anti-actin (1:1,000; Cytoskeleton; catalog #AAN02-S; RRID, AB_2884962) and goat polyclonal anti-mouse IgG H&L (1:20,000; Abcam catalog #ab205719; RRID, AB_2755049) antibodies were used for Western blot. Membranes were developed with Clarity Western ECL Substrate and imaged in a ChemiDoc Touch Imaging System (Bio-Rad Laboratories) for chemiluminescence. Western blot images were then quantified using the Image Lab software (v6.1.0; Bio-Rad Laboratories) by comparing the globular fraction's signal with each sample's filamentous fraction (G/F ratio).

### Human iPSC-derived sensory neurons

The human-induced pluripotent stem cell (hiPSC) line was manufactured and characterized at Anatomic Incorporated facilities with informed consent, using proprietary technologies. hiPSCs were differentiated into sensory neurons using a commercially available kit, Senso-DM, according to the manufacturer’s instructions (Anatomic Incorporated, #7007). Sensory neurons were maintained in culture at 37°C with 5% CO2 on glass coverslips coated with poly-l-lysine and Matrix 3 (Anatomic Incorporated, #M8003) in Senso-MM media (Anatomic Incorporated, #7008). hiPSC-derived sensory neurons were incubated in culture for 14 d before recording. Neurons were incubated in media supplemented with 5 µM SZ-3 for 18 h before electrophysiology experiments.

### Hippocampal neurons

Hippocampal neurons were dissected from early postnatal day (P1–P4) *Ube3a^m–/p+^* mice, either sex (Carrillo et al., 2020, 2021). The mice were decapitated, the skin and skull were removed, and the brain was placed in 4–5 ml ice-cold dissection solution (Earle's buffered salt solution, Invitrogen, 10 mm HEPES). Under a dissecting microscope, the hippocampus was removed and transferred to a solution containing 10 U/ml papain (Worthington), 20 µl DNase I (New England Biolabs, 2,000 U/mg), and 50 µM APV (Abcam) and incubated in a 37°C water bath for 30 min. Every 10 min, the tissue was pipetted to dissociate into individual cells. The activity of papain was stopped by the addition of 100% inactivated FBS for 2 min. The tissue was centrifuged and transferred to a dissociation solution containing B27/Neurobasal culture medium (Invitrogen), supplemented with 2 mM l-glutamine, 0.2% penicillin–streptomycin, and 20 mM glucose. The cell suspension was diluted with incubated feeding medium (B27/Neurobasal culture medium, supplemented with 2 mM l-glutamine, and 0.2% penicillin–streptomycin), plated on poly-l-lysine and laminin (Cell Applications)-coated glass coverslips, and incubated in 5% CO2 at 37 °C. Neurons were used 24 h after plating. Those used in experiments with SZ-3 (5 µM) compound were incubated for 3 h before recording.

### Electrophysiology

Patch-clamp recordings were performed on cells plated on glass coverslips. For whole-cell recordings of mechano-activated currents in the voltage-clamp mode, the bath solution contained (in mM) 140 NaCl, 6 KCl, 2 CaCl_2_, 1 MgCl_2_, 10 glucose, and 10 HEPES, pH 7.4, while the pipette solution contained (in mM) 140 CsCl, 5 EGTA, 1 CaCl_2_, 1 MgCl_2_, and 10 HEPES, pH 7.2. For current-clamp recordings of action potentials elicited by mechanical stimulation, the bath solution contained (in mM) 140 NaCl, 6 KCl, 2 CaCl_2_, 1 MgCl_2_, 10 glucose, and 10 HEPES, pH 7.4, while the pipette solution contained (in mM) 140 KCl, 6 NaCl, 2 CaCl_2_, 1 MgCl_2_, 10 glucose, and 10 HEPES, pH 7.4. Mechanical stimulation was performed using the voltage-clamp (constant −60 mV) or current-clamp configuration. Mechano-activated currents were sampled at 100 kHz and low-pass filtered at 10 kHz using a MultiClamp 700 B amplifier and Clampex (v10.4.2.0; Molecular Devices). Leak currents, before mechanical stimulation, were subtracted offline from the current traces, and data were digitally filtered at 2 kHz with ClampFit (v10.4.2.0; Molecular Devices). Recordings with leak currents >200 pA, with access resistance >10 MΩ, and cells with giga-seals that did not withstand at least five consecutive steps of mechanical stimulation were excluded from analyses.

For AMPA receptor recordings of hippocampal neurons, measurements were performed with borosilicate pipettes with a resistance between 8 and 15 mΩ, filled with internal solution as follows (in mM): 135 CsF, 33 CsCl, 2 MgCl_2_, 1 CaCl_2_, 11 EGTA, 10 HEPES, 0.4 GTP-Na, 4 ATP-Mg, and 5 phosphocreatine, pH 7.3. The bath solution contained 150 mM NaCl, 6 mM KCl, 2 mM CaCl_2_, 1 mM MgCl_2_, 10 mM HEPES and 10 mM glucose, 50 mM APV, 10 µM bicuculline, and 1 mM TTX, pH 7.4. The external solutions were locally applied to neurons using a SF-77B perfusion Fast-Step (Warner Instruments). All hippocampal neuron recordings were performed at room temperature with a holding potential of −60 mV using an Axopatch 200B amplifier, acquired at 10 kHz using pCLAMP10.7 software, and filtered online at 5 kHz.

For NMDA receptor currents, in transfected HEK293 cells, the pipette solution was comprised of (in mM) 135 CsF, 33 CsOH, 2 MgCl_2_, 1 CaCl_2_, 11 EGTA, and 10 HEPES, adjusted to pH 7.4 with CsOH. The bath solution contains the following (in mM): 140 NaCl, 2.8 KCl, 1 CaCl_2_, and 10 HEPES, adjusted to pH 7.4 with NaOH. The 1 mM glutamate and 1 mM glycine were applied to lifted cells using a stepper motor system (SF-77B, Warner Instruments) with triple-barrel tubing. For ASICs currents, in transfected HEK293 cells, the pipette solution was comprised of (in mM) 135 CsF, 33 CsOH, 2 MgCl_2_, 1 CaCl_2_, 11 EGTA, and 10 HEPES, adjusted to pH 7.4 with CsOH. The bath solution contains the following (in mM): 150 NaCl, 20 HEPES, 1 CaCl_2_, and 1 MgCl_2_ adjusted to pH 7.4, 6.0, or 5.0. Recordings were performed using an Axopatch 200B amplifier at −60 mV hold potential, acquired at 10 kHz using pCLAMP10 software, and filtered online at 5 kHz.

For TRP channels, transfected HEK293 cells, the bath solution contained (in mM) 140 NaCl, 6 KCl, 1 MgCl_2_, 10 glucose, and 10 HEPES, pH 7.4, while the pipette solution contained (in mM) 140 CsCl, 5 EGTA, and 10 HEPES, pH 7.2. Recordings were sampled at 100 kHz and low-pass filtered at 10 kHz using a MultiClamp 700 B amplifier and Clampex.

Current densities were calculated by dividing the current value (in picoamperes) by the measured cell membrane capacitance (picofarads). Before recording, cells were incubated with SZ-3 (5 or 10 µM for 18 h) or a control solution (DMSO alone), as indicated in each subsection of the methods.

### Mechanical stimulation

For indentation assays, DRG neurons, MCC13, and hiPSC-derived sensory neurons were mechanically stimulated with a heat-polished blunt glass pipette (3–4 µm) driven by a piezo servo controller (E625, Physik Instrumente). The blunt pipette was mounted on a micromanipulator at a ∼45° angle and positioned 3–4 µm above the cells. Displacement measurements were obtained with a square-pulse protocol consisting of 1 µm incremental indentation steps, each lasting 200 ms with a 2 ms ramp in 10 s intervals. The threshold of mechano-activated currents for each experiment was defined as the indentation step that evoked the first current deflection from the baseline. For current-clamp experiments, the mechanical threshold was defined as the indentation step that produced the first action potential. Only cells that did not detach throughout the stimulation protocols were included in the analyses. The piezo servo controller was automated, using a MultiClamp 700B amplifier, through Clampex (Molecular Devices).

### AFM

Atomic force microscopy (AFM) experiments were carried out with a BioScope II Controller (Bruker), operated with the Research NanoScope software version 7.30 integrated to a Nikon TE2000-E inverted optical microscope (Nikon Instruments) to facilitate bright-field/fluorescence imaging. Bead sphere colloid cantilever probes (Novascan PT.GS) consisted of a 5-µm-diameter borosilicate glass particle attached to the edge of a silicon nitride V-shaped cantilever with a nominal spring constant of 0.24 N/m. Each cantilever is calibrated for its laser sensitivity using the thermal oscillation method before each experiment. Indentation curves were captured using a 4 µm ramp size, at a scan rate of 0.5 Hz, and a trigger threshold with a maximum load of 10 nN. Young's modulus values were calculated following the Hertz model (spherical indenter radius, 2.5 µm) with a Poisson's ratio of 0.5, using the NanoScope Analysis software version 3.0 (Copyright Bruker).

### Intraperitoneal injections

Cofilin inhibitor was dissolved in a vehicle solution that consisted of 4.9% DMSO, 4.9% Tween 20, and 88.9% 2-hydroxypropyl-β-cyclodextrin, yielding a final concentration of 1.3%. *Ube3a^m–/p+^* mice between the ages of 3 and 4 months were administered SZ-3 or vehicle for 10 d, at the same time each day by a technician at the UT Health mouse facility. For T-maze behavioral experiments, mice received SZ-3 at a dose of 10 mg/kg. For rotarod and von Frey assays, a dose of 25 mg/kg was administered.

### Behavior

For rotarod, mice were acclimated to the experimental room for 1 h before the test. Mice were trained for 3 d by placing them on a rod (SDI rotor-rod, San Diego Instruments) that rotated at a constant speed of 4 rpm for 2 min. On the test day, mice were placed on the rod, which accelerated from 4 to 40 rpm in 5 min, and their latency to fall was recorded. Three trials were taken of each mouse with a 15 min interval between each trial. The average of three trials was used for analysis. The baseline behavior was recorded before the mice were administered the drug or vehicle and after 10 d of intraperitoneal injections. For T-maze, testing was performed by placing a mouse on the lower end of the T-maze and allowing it to choose one side spontaneously. After the initial choice, they are closed off with an insert to let them explore the arm of their choice for 30 s. Afterward, they are put back in their home cages for ∼2–3 min. Meanwhile, the researcher wipes the maze down with a paper towel to avoid odor queues and clean feces or urine. Then, mice are placed back in the start arm of the T-maze and tasked to make another choice. Choosing the same arm of the maze was counted as a failed alternation. If they choose the opposite arm, this was counted as a successful alternation. Each mouse repeated this protocol four times. Each mouse was habituated to the maze for 5 min the day before testing. Testing was performed on Day 7 and Day 12. For von Frey, mice were acclimated to the behavioral testing room and individually placed in modular holding chambers constructed from 3-mm-thick PVC positioned atop a metal mesh platform. Following a 1 h habituation period, von Frey filaments were applied to the plantar surface of both hindpaws in an ascending order of force. Each filament was applied three times per paw. A positive response, defined as a clear withdrawal or licking of the paw, was recorded if it occurred in at least two of the three applications. If no positive response was elicited, the next filament of greater force was applied. The paw withdrawal threshold was defined as the minimal force that evoked a positive response in two out of three trials. Final threshold values were reported as the average of two independent measurements taken from both the left and right hindpaws.

### Data analysis

Data were plotted using OriginPro (2018 v:b9.51.195; OriginLab) and Estimation Stats (Ho et al., 2019). The time constant of inactivation τ was obtained by fitting a single exponential function between the peak value of the current at the end of the stimulus as follows:

$$f_{\left(t\right)}=\;\overset{n}{\underset{i=1}{\sum }}\;A_{i}\;*\;e^{\frac{-t}{\tau_{i}}}+C,$$

where *A* is the amplitude, *τ* is the time constant, and *C* is the constant *y*-offset for each component *i*. All boxplots show mean (square), median (bisecting line), bounds of box (75th to 25th percentiles), and outlier range with 1.5 coefficient (whiskers). Statistical analyses were performed using the GraphPad InStat software (version 3.10; GraphPad Software) and Estimation Stats (Ho et al., 2019). We used the Kolmogorov and Smirnov method to determine data distribution, as well as the Bartlett’s test to determine differences between standard deviations. Individual tests are described in each of the figure legends.

### Statistical analysis

No statistical method was used to predetermine the sample size, and no data were excluded from the analyses. The experiments were not randomized. For electrophysiology, the investigator was blind to genotype and treatment. For behavioral assays, the investigators were blind when possible. All attempts at replication were successful. Experiments were performed at least three times on different days with different/independent preparations.

### Data availability

The source data underlying figures are provided as a Source Data file and deposited at https://doi.org/10.6084/m9.figshare.28908632.

## Results

### Genetic manipulation of cofilin modulates PIEZO2 function

**Figure 1.**
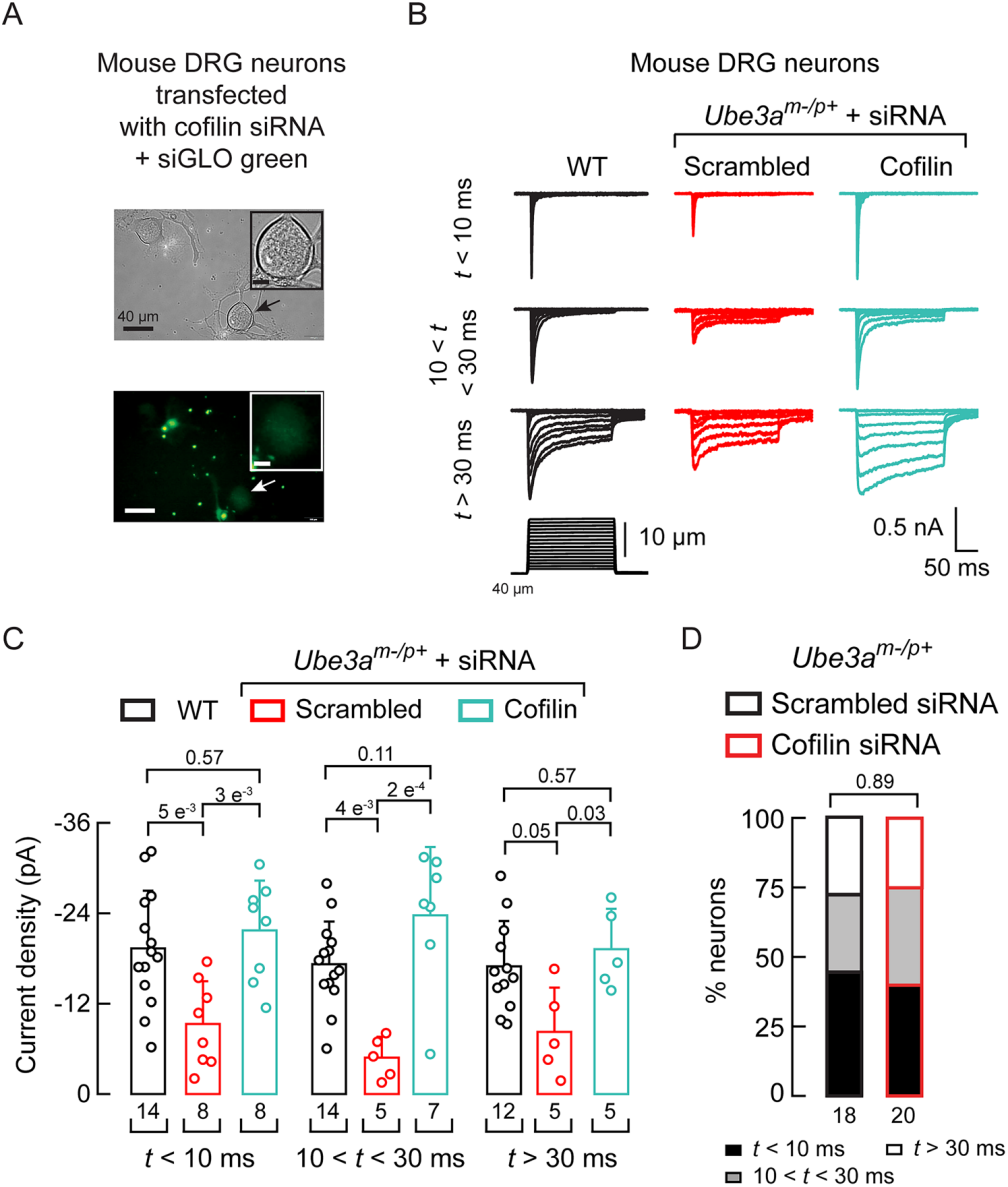
Knocking down cofilin increases PIEZO2 function. ***A***, Micrographs showing mouse *Ube3a^m–/p+^* DRG neurons (top) transfected with cofilin siRNA and the transfection marker siGLO green (bottom). Micrographs are representative of at least 10 independent preparations. Black and white arrows highlight a positively transfected neuron. ***B***, Representative whole-cell patch–clamp recordings elicited by mechanical stimulation (−60 mV) of rapidly, intermediate, and slowly inactivating currents of *Ube3a^m–/p+^* DRG neurons transfected with scrambled or cofilin siRNAs. ***C***, Current densities elicited by maximum displacement of *Ube3a^m–/p+^* DRG neurons, transfected with scrambled or cofilin siRNAs, classified by their time constant of inactivation. Error bars indicate mean ± SD. Kruskal–Wallis (*H* = 31.45; *p* = 0.00012) and Dunn’s multiple-comparison test. ***D***, Proportions of *Ube3a^m–/p+^* DRG neurons, transfected with scrambled or cofilin siRNA, classified by their time constant of inactivation of mechano-activated currents elicited by maximum displacement. *χ*^2^ test (*χ*^2^ = 0.23; *p* = 0.89). *n* is denoted in each panel. Post hoc *p* values are denoted in the corresponding panels.

We demonstrated that the loss of *UBE3A* expression leads to increased cofilin levels and decreased PIEZO2 currents (Romero et al., 2023). Here, we used mice carrying a LOF *Ube3a* mutation on the maternal allele in a C57BL/6 genetic background (Jiang et al., 1998). Heterozygous mice with a maternal deficiency (*Ube3a^m–/p+^*) exhibit AS-associated phenotypes, including a lack of balance and coordination (Jiang et al., 1998; Sonzogni et al., 2018). We aimed to determine whether reducing cofilin expression through siRNA-mediated silencing in DRG neurons from *Ube3a^m–/p+^* mice could restore mechanocurrents (Fig. 1*A*). Our results showed that all mechanocurrents, including the rapidly inactivating currents associated with PIEZO2, were significantly enhanced after cofilin knockdown compared with scrambled siRNA controls (Fig. 1*B*,*C*). The distribution of DRG neurons exhibiting rapidly inactivating, intermediate, and slow mechanocurrents was similar in neurons treated with scrambled and cofilin siRNA (Fig. 1*D*).

### Optogenetic activation of cofilin decreases PIEZO2 currents

**Figure 2.**
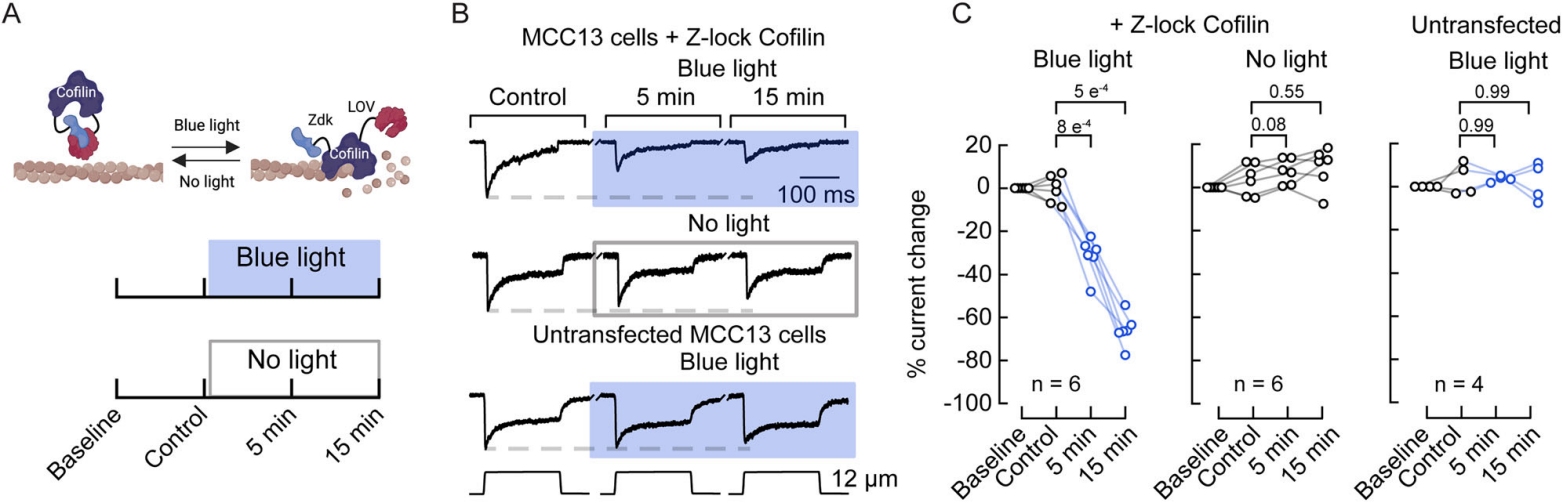
Cofilin photoactivation decreases mechanosensitivity in MCC13 cells. ***A***, Top, diagram describing the photoswitchable *Z*-lock cofilin. The light oxygen voltage (LOV) domain and Zdk, a small protein that selectively binds LOV in the dark, are appended to cofilin, where they sterically block the active site. Made with BioRender.com. Bottom, Timeline depicting the experimental design for modulation of mechanoresponses by photoswitchable *Z*-lock cofilin. ***B***, Representative whole-cell patch–clamp recordings elicited by mechanical stimulation (12 µm; −60 mV) of MCC13 cells untransfected or transfected with *Z*-lock cofilin and recorded in the dark or after exposure to blue light (450 nm) as described in ***A***. ***C***, Left, current changes elicited by 12 µm displacement of MCC13 cells transfected with *Z*-lock cofilin exposed to blue light. Data samples are paired. Repeated-measure ANOVA (*F* = 159.55; *p* = 1.33^−11^) with Tukey–Kramer multiple-comparison test. Middle, Current changes elicited by 12 µm displacement of MCC13 cells transfected with *Z*-lock cofilin in the dark. Right, Current changes elicited by 12 µm displacement of MCC13 cells untransfected, exposed to blue light. Data samples are paired. Repeated-measures ANOVA (*F* = 3.92; *p* = 0.029) with Tukey–Kramer multiple-comparison test. *n* is denoted in each panel. Post hoc *p* values are denoted in the corresponding panels.

To assess whether the effect of cofilin could also occur acutely, we utilized an optogenetic approach to rapidly modulate its actin-severing ability with light using the *Z*-lock cofilin construct (Stone et al., 2019). In dark conditions, the interaction of Zdk (a protein A fragment) with the LOV2 domain of *Avena sativa* phototropin 1, linked to the C- and *N*-termini of cofilin, occludes the active site of cofilin (Stone et al., 2019). Blue light irradiation (400–500 nm) disrupts this interaction, exposing cofilin’s active site (Fig. 2*A*, top panel). We measured mechanocurrents using the whole-cell patch–clamp configuration from MCC13 cells that were transiently transfected with *Z*-lock cofilin. Measurements were taken in the dark at 0, 5, and 15 min and after exposure to blue light for 5 and 15 min **(**450 nm; Fig. 2*A*, bottom panel). Importantly, we observed a time-dependent reduction in mechanocurrent amplitudes following the photoactivation of *Z*-lock cofilin (Fig. 2*B*,*C*). Control experiments in cells lacking *Z*-lock cofilin showed no effect from blue light exposure, confirming that illumination alone did not affect mechanoresponses (Fig. 2*B*,*C*). Our findings indicate that acute cofilin activation decreases mechanocurrents.

### A first-in-class cofilin inhibitor enhances PIEZO2 currents

**Figure 3.**
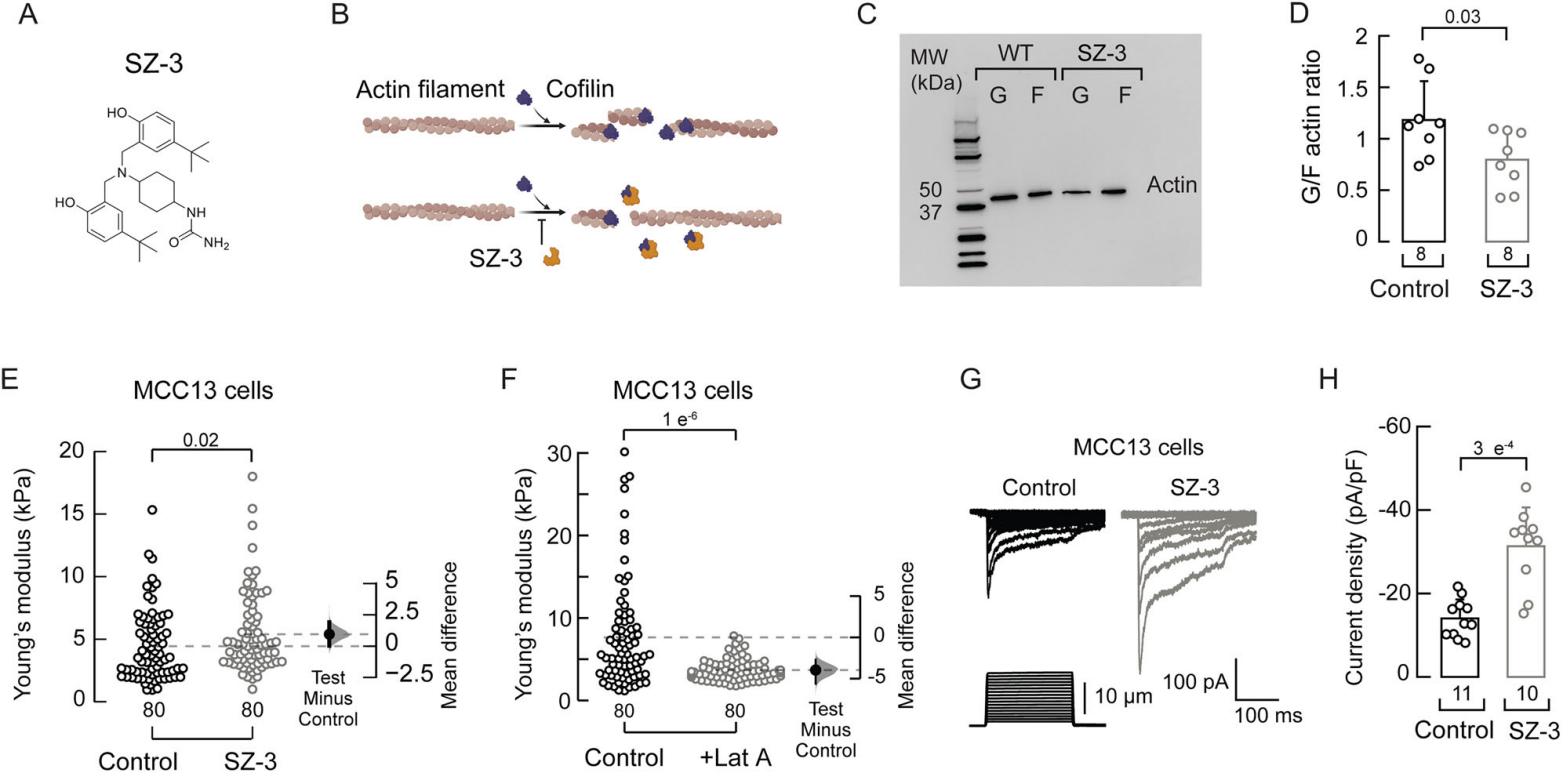
SZ-3 increases cell stiffness and mechanosensitivity in MCC13 cells. ***A***, Chemical representation of the cofilin inhibitor SZ-3. ***B***, Diagram describing the effect of cofilin inhibition by SZ-3 on actin filaments. Made with BioRender.com. ***C***, Western blot of soluble and insoluble actin (G and F, respectively) of control (DMSO) or SZ-3 (10 µM)-treated MCC13 cells. ***D***, G-/F-actin ratios. Error bars indicate mean ± SD. Two-tailed unpaired *t* test (*t* = 2.34). ***E***, Young’s modulus data points are presented as a swarmplot for control (DMSO) or SZ-3 (10 µM)-treated MCC13 cells. Two-tailed Mann–Whitney test (*U* = 2,509.5). ***F***, Young’s modulus data points are presented as a swarmplot for control (EtOH) and latrunculin A-treated MCC13 cells. Two-tailed Mann–Whitney test (*U* = 4,596.5). ***G***, Representative whole-cell patch–clamp recordings elicited by mechanical stimulation (−60 mV) of control (DMSO) or SZ-3 (10 µM)-treated MCC13 cells. ***H***, Current densities elicited by maximum displacement of control (DMSO) or SZ-3 (10 µM)-treated MCC13 cells. Error bars indicate mean ± SD. Two-tailed Mann–Whitney test (*U* = 7). *n* is denoted in each panel. Post hoc *p* values are denoted in the corresponding panels.

Since we observed an inverse relationship between cofilin expression and PIEZO2 function (Figs. 1, 2), we hypothesized that cofilin inhibition could enhance mechanocurrents and compensate for the mechanosensory deficits caused by the loss of *UBE3A* expression in AS. Recently, we developed SZ-3, a first-in-class small–molecule cofilin inhibitor (Fig. 3*A*,*B*; Alaqel et al., 2022). MCC13 cells treated with SZ-3 displayed a reduced G-/F-actin ratio (i.e., more F-actin), as confirmed by Western blots (Fig. 3*C*,*D*). AFM further demonstrated that SZ-3 treatment increases the Young’s modulus (i.e., stiffness) of MCC13 cells compared with the control (DMSO; Fig. 3*E*). On the other hand, treatment of MCC13 with latrunculin A, which disrupts F-actin, resulted in a decrease in the Young’s modulus compared with the control **(**EtOH; Fig. 3*F***)**. The increased F-actin and Young’s modulus observed with SZ-3 treatment may enhance mechanosensitive channel function. Indeed, MCC13 cells challenged with SZ-3 exhibit larger mechanocurrents than under control conditions (Fig. 3*G*,*H*).

**Figure 4.**
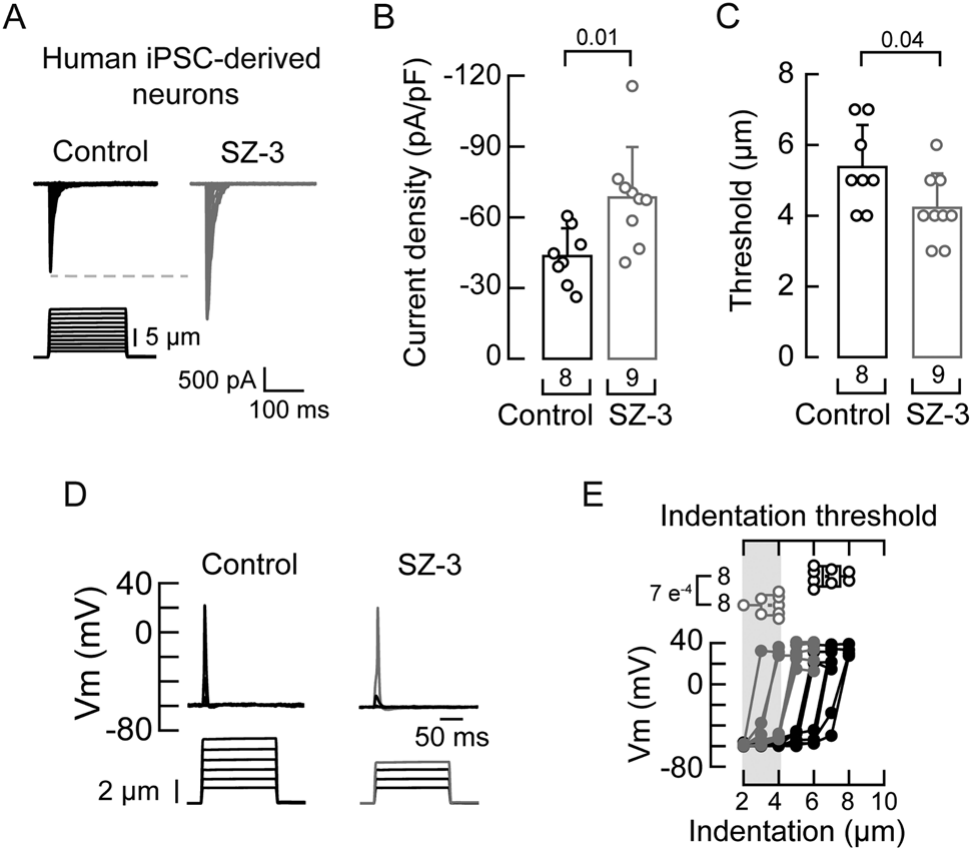
Cofilin inhibition increases PIEZO2 function and mechanoexcitability in human-derived sensory neurons. ***A***, Representative whole-cell patch–clamp recordings of currents elicited by mechanical stimulation (−60 mV) of control (DMSO) or SZ-3 (5 µM)-treated iPSC–derived sensory neurons. ***B***, Current densities elicited by maximum displacement of control (DMSO) or SZ-3 (5 µM)-treated iPSC–derived neurons. Error bars indicate mean ± SD. Two-tailed unpaired *t* test (*t* = 2.92). ***C***, Boxplots show the displacement thresholds required to elicit mechanocurrents in control (DMSO) or SZ-3 (5 µM)-treated iPSC–derived neurons. Two-tailed unpaired *t* test (*t* = 2.2). ***D***, Current-clamp recordings of membrane potential changes elicited by indentation of control (DMSO) or SZ-3 (5 µM)-treated iPSC-derived neurons. ***E***, Membrane potential peak versus mechanical indentation of independent iPSC-derived neurons. At the top, boxplots show the displacement threshold required to elicit an action potential in these neurons. Two-tailed Mann–Whitney test (*U* = 0). *n* is denoted in each panel. Post hoc *p* values are denoted in the corresponding panels.

To extend our findings to a human neuronal model, we utilized hiPSC-derived sensory neurons generated using the Anatomic Incorporated differentiation kit. In an earlier study, we demonstrated that these in vitro-derived sensory neurons display mechanocurrents characteristic of PIEZO2 (Romero et al., 2023). In these neurons, we also found that SZ-3 enhances PIEZO2 currents while decreasing the displacement threshold (Fig. 4*A*–*C*). We have previously shown that DRG neurons from *Ube3a^m–/p+^* mice exhibit reduced mechanoexcitability (Romero et al., 2023). Hence, we tested whether SZ-3 could enhance mechanoexcitability in hiPSC-derived sensory neurons. Indeed, neurons treated with SZ-3 required smaller indentation steps (≤6 µm) to elicit mechanically activated action potentials compared with untreated neurons (Fig. 4*D*,*E*). Our results demonstrate that SZ-3 increases cell stiffness, mechanocurrents, and PIEZO2 function as well as enhances mechanical excitability in hiPSC-derived sensory neurons.

### SZ-3 recovers *Ube3a^m–/p+^* neuronal mechanocurrents and mechanoexcitability

**Figure 5.**
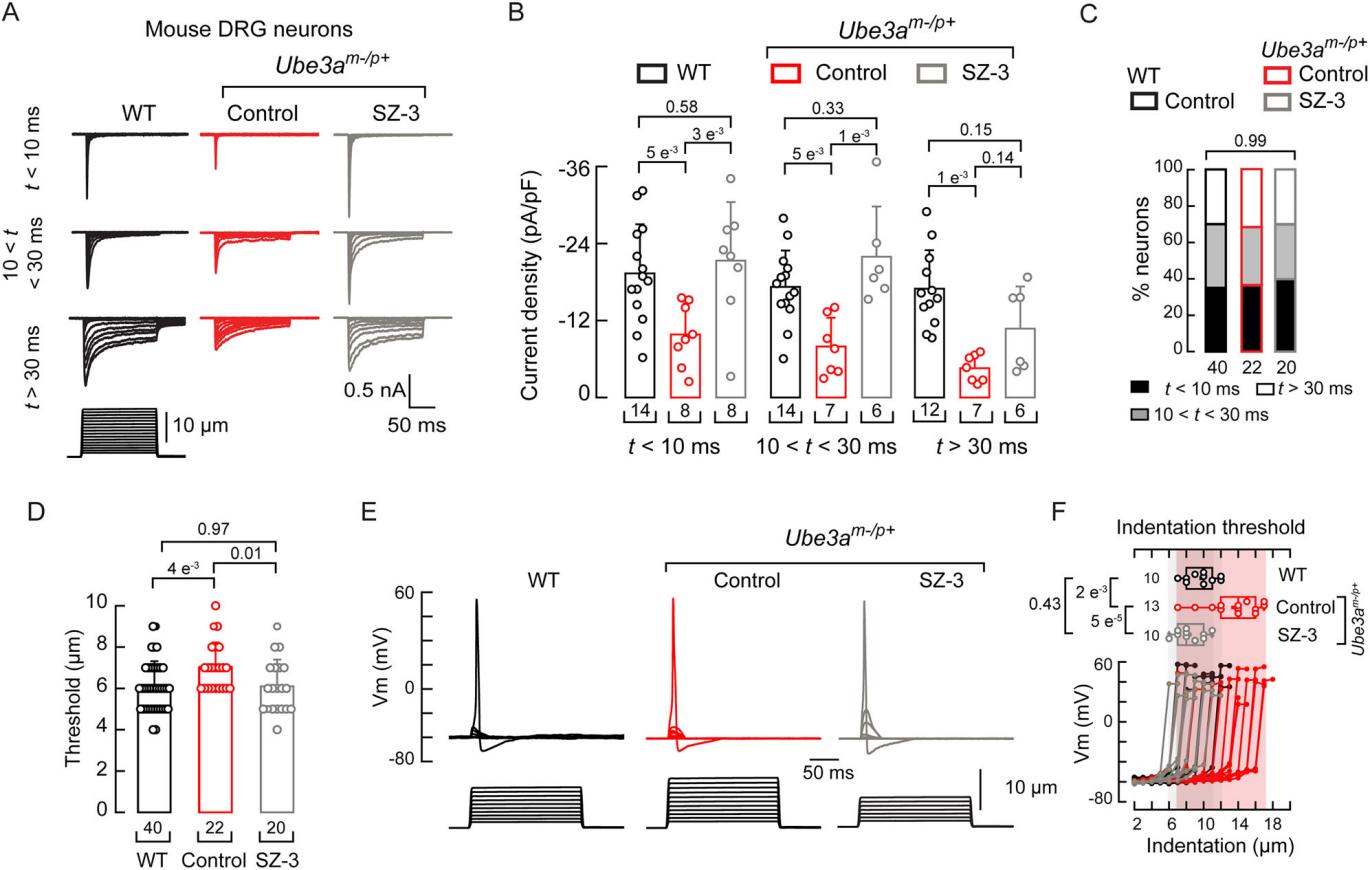
SZ-3 treatment increases *Ube3a^m–/p+^* mouse mechanocurrents and mechanoexcitability. ***A***, Representative whole-cell patch–clamp recording elicited by mechanical stimulation (−60 mV) of rapidly, intermediate, and slowly inactivating currents of WT, control (DMSO) or SZ-3 (5 µM)-treated *Ube3a^m–/p+^* DRG neurons. ***B***, Current densities elicited by maximum displacement of DRG neurons, classified by their time constant of inactivation. Error bars indicate mean ± SD. Kruskal–Wallis (*H* = 38.71; *p* = 5.56^−6^) and Dunn’s multiple-comparison test. ***C***, Proportions of WT, control (DMSO), or SZ-3 (5 µM for 18 h)-treated *Ube3a^m–/p+^* DRG neurons classified by their time constant of inactivation of mechano-activated currents elicited by maximum displacement. *χ*^2^ test (*χ*^2^ = 0.22; *p* = 0.99). ***D***, Displacement thresholds required to elicit mechanocurrents in WT, control (DMSO), or SZ-3(5 µM)-treated *Ube3a^m–/p+^* DRG neurons. Error bars indicate mean ± SD. Kruskal–Wallis (*H* = 9.25; *p* = 0.0098) and Dunn’s multiple-comparison test. ***E***, Representative current-clamp recordings of membrane potential changes elicited by mechanical stimulation of WT, control (DMSO), or SZ-3 (5 µM)-treated *Ube3a^m–/p+^* DRG neurons. ***F***, Membrane potential peak versus mechanical indentation of independent WT, control (DMSO), or SZ-3 (5 µM)-treated *Ube3a^m–/p+^* DRG neurons. At the top, boxplots show the displacement threshold required to elicit an action potential in these neurons. Boxplots show mean (square), median (bisecting line), bounds of box (75th to 25th percentiles), outlier range with 1.5 coefficient (whiskers), and minimum and maximum data points. One-way ANOVA (*F* = 14.52; *p* = 3.87^−5^) with Tukey’s multiple-comparison test. *n* is denoted in each panel. Post hoc *p* values are denoted in the corresponding panels.

We previously showed that, in a mouse model of AS (*Ube3a^m–/p+^*), DRG neurons exhibit reduced mechanocurrents and mechanoexcitability (Romero et al., 2023). Cultured *Ube3a^m–/p+^* mouse DRG neurons challenged with SZ-3 displayed increased mechanocurrents (including the rapidly inactivating currents assigned to PIEZO2) that are reminiscent of those from wild-type (WT) littermate neurons (Fig. 5*A*,*B*). The SZ-3 treatment did not affect the distribution of DRG neurons exhibiting rapidly inactivating, intermediate, and slow mechanocurrents (Fig. 5*C*). Moreover, the displacement threshold required to elicit mechanocurrents in the *Ube3a^m–/p+^* neurons was lower after SZ-3 treatment compared with the control and similar to the DRG neurons of WT mice (Fig. 5*D*). Similarly, DRG neurons from *Ube3a^m–/p+^* mice, in the presence of SZ-3, required smaller indentation steps (≤10µm) to trigger action potentials, akin to WT, whereas untreated neurons required larger indentations (≥12 µm; Fig. 5*E*,*F*). Overall, we demonstrate that stabilizing F-actin with SZ-3 restores mechanocurrents and mechanoexcitability in DRG neurons of an AS mouse model.

### SZ-3 increases glutamate-dependent currents in *Ube3a^m–/p+^* hippocampal neuron cultures

**Figure 6.**
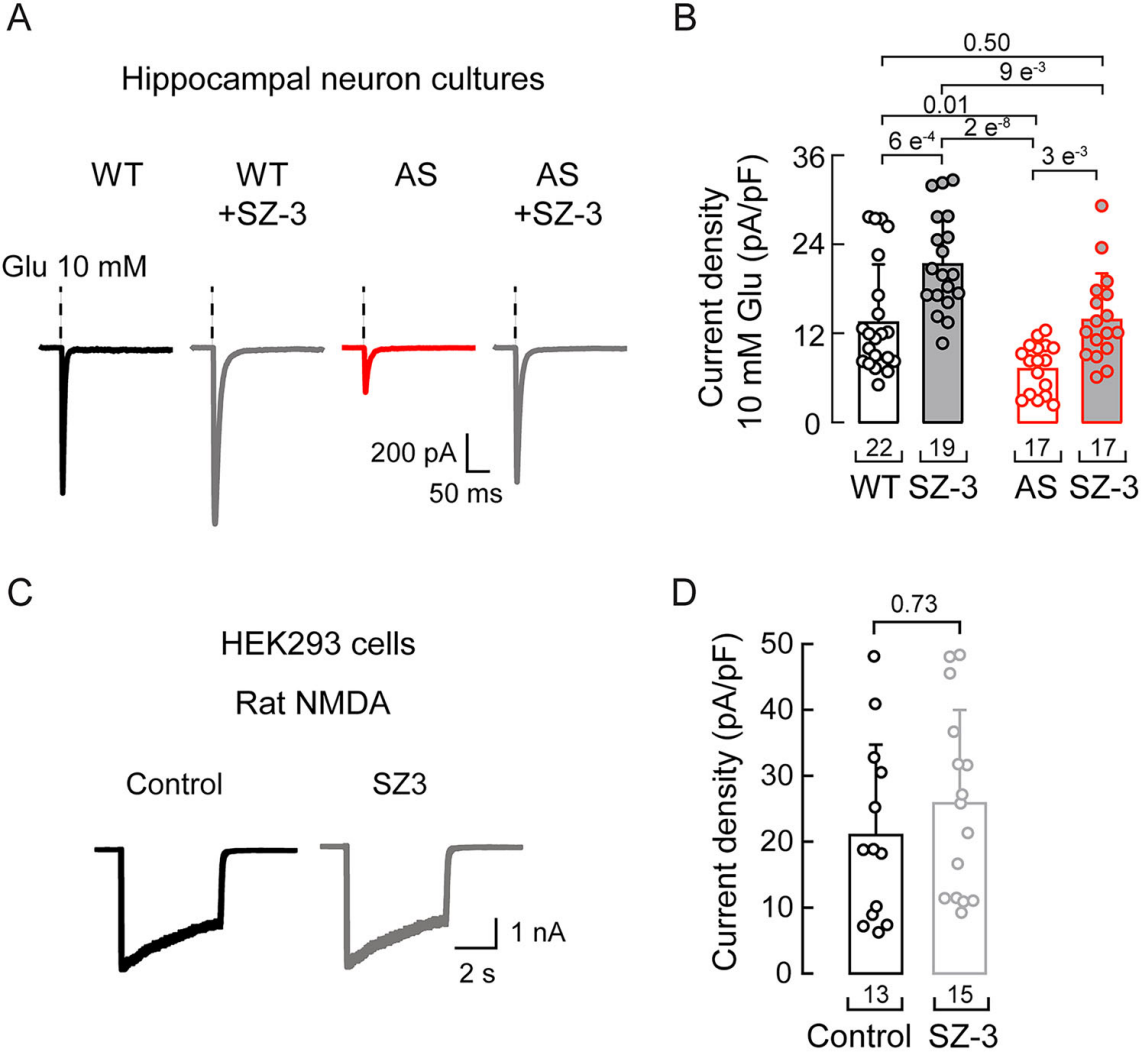
SZ-3 increases glutamate-activated currents in hippocampal neurons from *Ube3a^m–/p+^* mice. ***A***, Representative traces of whole-cell patch–clamp recordings, elicited by application of 10 mM glutamate in control (vehicle) or SZ-3 (5 µM; 3 h)-treated primary hippocampal neurons from WT or *Ube3a^m–/p+^* mice. ***B***, Current densities of control (vehicle) or SZ-3 (5 µM; 3 h)-treated primary hippocampal neurons from WT or *Ube3a^m–/p+^* mice, elicited by 10 mM glutamate. Kruskal–Wallis (*H* = 32.4; *p* = 4.33^−8^) and Dunn’s multiple-comparison test. Error bars indicate mean ± SD. ***C***, Representative whole-cell patch–clamp recordings, elicited by glutamate and glycine (1 mM), of NMDA receptors transfected in HEK293 cells treated with control (DMSO) or SZ-3 (5 µM). ***D***, Current densities, elicited by glutamate and glycine (1 mM), of NMDA receptors transfected in HEK293 cells treated with control (DMSO) or SZ-3 (5 µM). Error bars indicate mean ± SD. Two-tailed Mann–Whitney test (*U* = 368; *p* = 0.726). *n* is denoted in each panel. Post hoc *p* values are denoted in the corresponding panels.

**Figure 7.**
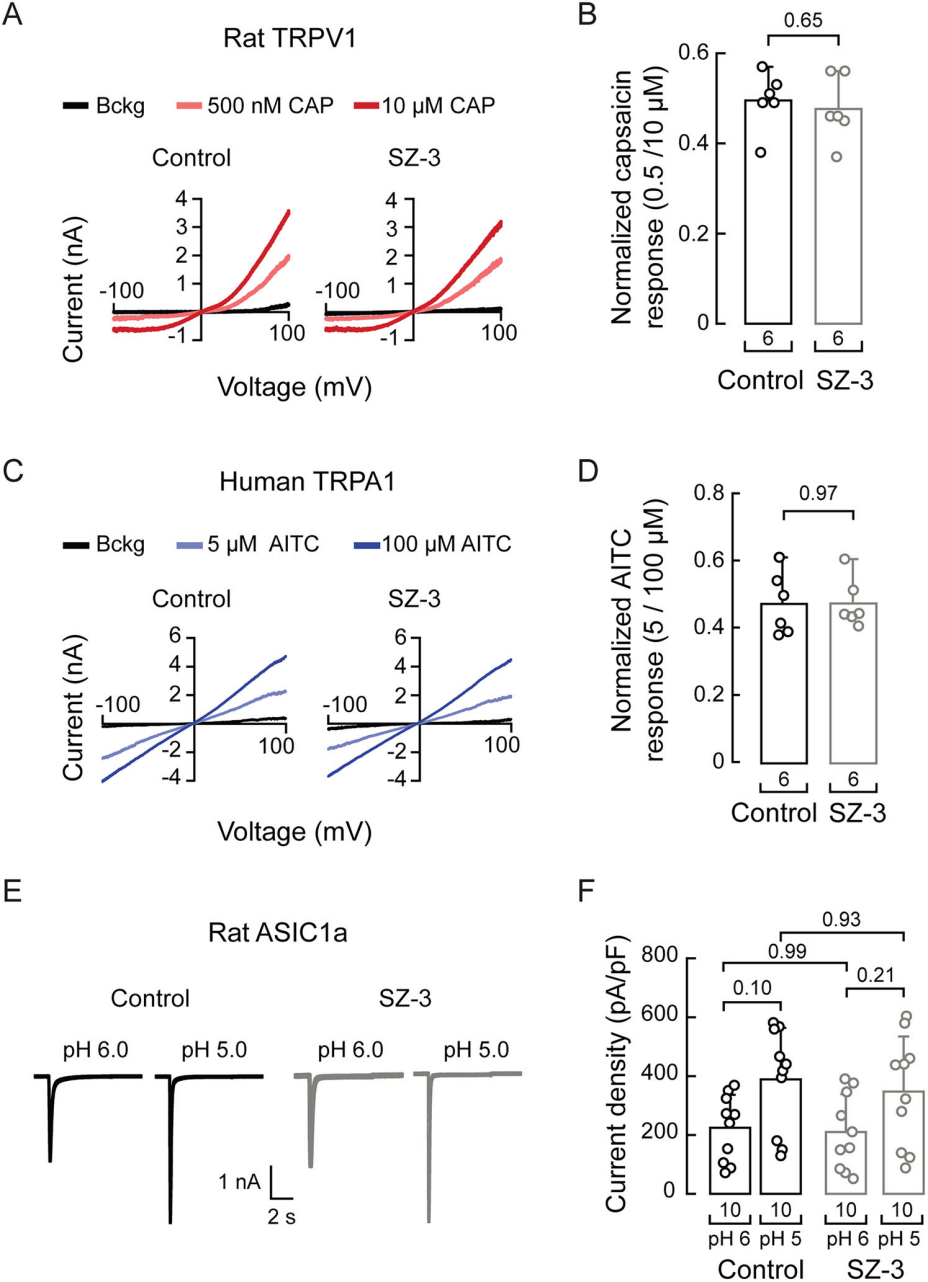
SZ-3 does not modulate TRPV1, TRPA1, or ASIC1a activity. ***A***, Representative whole-cell patch–clamp recordings elicited by capsaicin (500 nM and 10 µM) of rTRPV1-transfected HEK293 cells treated with control (DMSO) or SZ-3 (5 µM). ***B***, Normalized capsaicin response (500 nM/10 µM) of rTRPV1-transfected HEK293 cells treated with control (DMSO) or SZ-3 (5 µM). Error bars indicate mean ± SD. Two-tailed unpaired *t* test (*t* = 0.464). ***C***, Representative whole-cell patch–clamp recordings elicited by AITC (5 µM and 100 µM) of hTRPA1-transfected HEK293 cells treated with control (DMSO) or SZ-3 (5 µM). ***D***, Normalized AITC response (5 µM/100 µM) of hTRPA1-transfected HEK293 cells treated with control (DMSO) or SZ-3 (5 µM). Error bars indicate mean ± SD. Two-tailed unpaired *t* test (*t* = −0.033). ***E***, Representative whole-cell patch–clamp recordings, elicited by acidic pH, of rASIC1a-transfected HEK293 cells treated with control (DMSO) or SZ-3 (5 µM). ***F***, Current densities, elicited by acidic pH, of rASIC1a-transfected HEK293 cells treated with control (DMSO) or SZ-3 (5 µM). Error bars indicate mean ± SD. Two-way ANOVA with Tukey’s multiple-comparison test (*F* = 0.078; *p* = 0.781). *n* is denoted in each panel. Post hoc *p* values are denoted in the corresponding panels.

AMPA receptors are expressed in hippocampal neurons, where they are essential for glutamate-dependent excitatory neurotransmission and spatial learning (Conboy and Sandi, 2010; Keifer and Zheng, 2010; Kamalova and Nakagawa, 2021; Yao et al., 2022). Previous work demonstrated that AMPA receptor currents are decreased in hippocampal neurons from a mouse model of AS (Greer et al., 2010). Importantly, Gu et al. showed that cofilin activity and actin dynamics influence AMPA receptor trafficking and function in rat hippocampal neurons (Gu et al., 2010). Therefore, we hypothesized that inhibiting cofilin activity may enhance AMPA function in the CNS, akin to PIEZO2 in the peripheral nervous system. To assess AMPA receptor function, we recorded AMPA currents in primary hippocampal neurons from *Ube3a^m–/p+^* mice and their WT littermates treated with SZ-3 (Fig. 6*A*,*B*) while inhibiting NMDA receptors (using Mg^2+^ and 2-amino-5-phosphonovaleric acid). We found that SZ-3 significantly enhanced glutamate-dependent AMPA currents (Fig. 6*A*,*B*). Control experiments in HEK293 cells transiently expressing NMDA receptors, TRPV1, TRPA1, and ASIC1a, demonstrated that SZ-3 did not alter their currents under similar experimental conditions (Fig. 6*C*,*D*; Fig. 7). These results suggest that SZ-3 does not have a broad effect on ion channel function. Together, our findings suggest that inhibiting cofilin activity may enhance AMPA receptor function in the context of AS.

### Cofilin inhibition improves *Ube3a^m–/p+^* mice rotarod and T-maze performances

**Figure 8.**
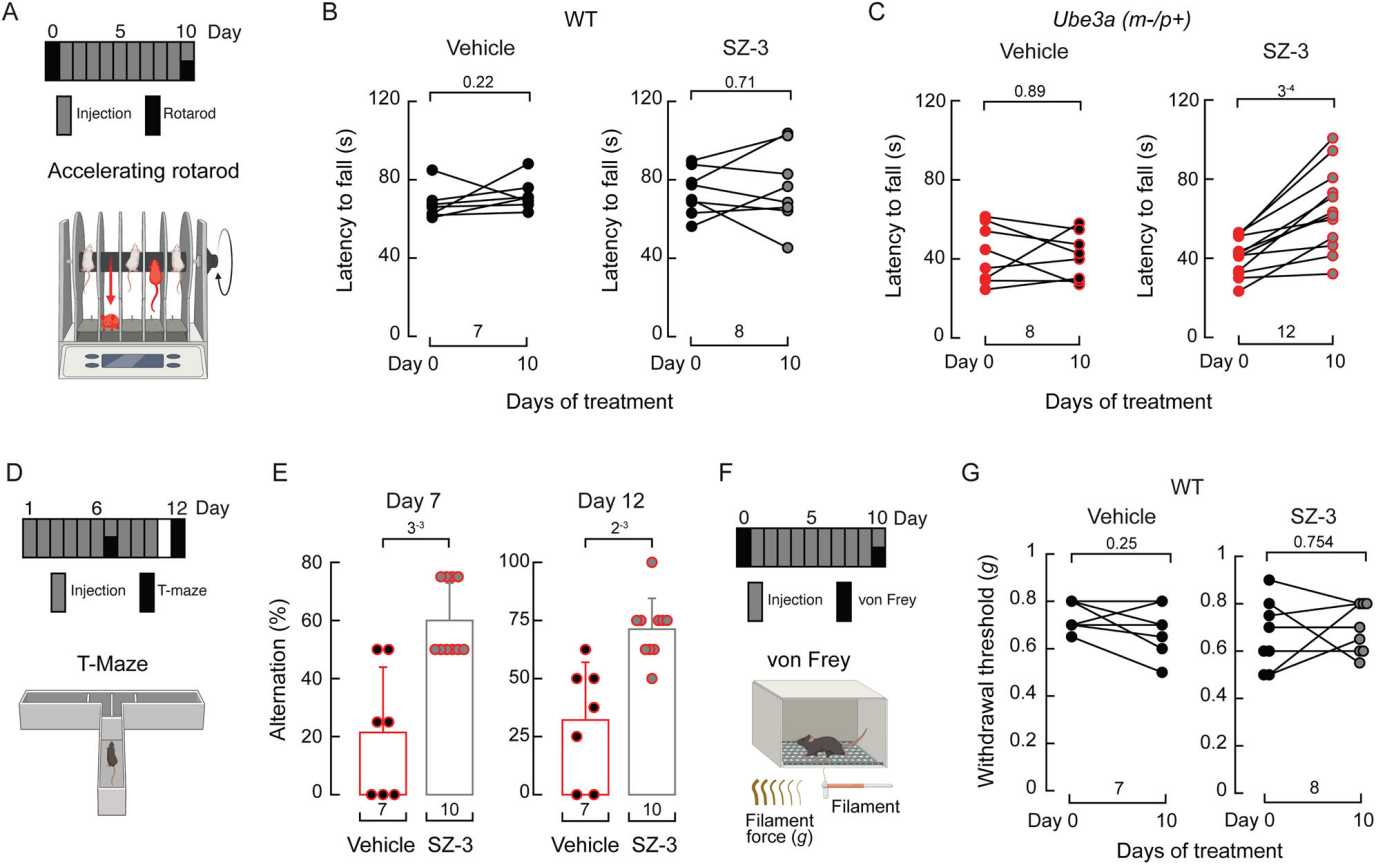
SZ-3 improves *Ube3a^m–/p+^* mouse rotarod and T-maze performance. ***A***, Diagram depicting the experimental timeline and rotarod behavioral assay. Made with BioRender.com. ***B***, Latency to fall changes before and after 10 d of vehicle or SZ-3 treatment of WT mice. Data samples are paired. Paired Wilcoxon signed-rank test for the vehicle group (*W* = 6). Two-tailed paired *t* test for the AZ-3 group (*t* = 0.392). ***C***, Latency to fall changes before and after 10 d of vehicle or SZ-3 treatment of *Ube3a^m–/p+^* mice. Data samples are paired. Two-tailed paired *t* test (*t* = 0.24 for vehicle and *t* = 5.22 for SZ-3). ***D***, Diagram depicting the experimental timeline and T-maze behavioral assay. Made with BioRender.com. ***E***, Percentage alternation at Day 7 of the vehicle or SZ-3 treatment of *Ube3a^m–/p+^* mice. Error bars indicate mean ± SD. Two-tailed Mann–Whitney test (*U* = 6). ***E***, Percentage alternation at Day 12 of the vehicle or SZ-3 treatment of *Ube3a^m–/p+^* mice. Error bars indicate mean ± SD. Two-tailed Mann–Whitney test (*U* = 3.5). ***F***, Diagram depicting the experimental timeline and the von Frey behavioral assay. Made with BioRender.com. ***G***, Withdrawal thresholds elicited with von Frey filaments before and after 10 d of vehicle or SZ-3 treatment of WT mice. Paired Wilcoxon signed-rank test for the vehicle group (*W* = 12.5). Two-tailed paired *t* test for the AZ-3 group (*t* = 0.326). *n* is denoted in each panel. Post hoc *p* values are denoted in the corresponding panels.

Individuals with AS and the corresponding mouse models exhibit impaired motor coordination and learning disabilities (Tan et al., 2011; Larson et al., 2015; Buiting et al., 2016; Wheeler et al., 2017). The accelerating rotarod is a widely used and reliable assay for evaluating rodent motor coordination (Jiang et al., 2010; Born et al., 2017; Sonzogni et al., 2018; Rotaru et al., 2020; Fig. 8*A*, bottom panel). While we previously found that a linoleic acid-enriched diet ameliorated gait ataxia in a mouse model of AS, it did not enhance rotarod performance. Since the recovery of peripheral mechanosensory function was insufficient to improve rotarod performance in AS mice (Romero et al., 2023), we hypothesized that inhibiting cofilin in the CNS might improve it. Hence, we administered daily intraperitoneal injections of SZ-3 or the vehicle for 10 consecutive days (Fig. 8*A*, top panel). Remarkably, SZ-3-treated *Ube3a^m—/p+^* mice exhibited improved rotarod performance compared with those receiving the vehicle treatment or WT mice treated with SZ-3 (Fig. 8*B*,*C*).

T-mazes are standard behavioral assays used to assess the cognitive abilities (e.g., spatial learning and memory) of rodents (Deacon and Rawlins, 2006; Fig. 8*D*, bottom panel). We have previously utilized this behavioral test and demonstrated that SZ-3 improves cognitive function in a mouse model (WT) of intracerebral hemorrhage (Almarghalani et al., 2024). Since T-maze alternation is sensitive to hippocampal dysfunction, we tested whether SZ-3 treatment could improve the performance of this task. Notably, after 7 consecutive days of SZ-3 injection, *Ube3a^m–/p+^* mice exhibited a significant increase in T-maze alternation (Fig. 8*D*,*E*). This improvement in spatial learning and memory persisted when the test was repeated 2 d after the 10 d treatment was completed (Fig. 8*E*). Control experiments in WT mice showed that SZ-3 did not affect touch sensitivity, as determined by von Frey assays (Fig. 8*F*,*G*). Overall, our results highlight the role of actin-binding proteins in motor and learning disabilities in the context of AS.

## Discussion

Our study sheds light on how cofilin modulates ion channel function, underscoring the therapeutic potential of targeting actin-binding proteins in AS. Here, we demonstrate that acute cofilin activation using optogenetics decreases mechanocurrents, while genetic and pharmacological (with the first-in-class cofilin inhibitor SZ-3) inhibition restores PIEZO2 currents and mechanoexcitability in AS. Additionally, we show that SZ-3 restores AMPA receptor function in cultured hippocampal neurons and enhances motor coordination and cognitive function in a mouse model of AS.

The cytoskeleton continuously regulates the function of membrane proteins, including ion channels (Janmey et al., 1998). Proper actin turnover and organization are critical for maintaining channel mobility and activity (Spence and Soderling, 2015). Thus, proteins that govern the dynamic assembly and disassembly of the actin cytoskeleton (e.g., cofilin) may influence channel function under basal conditions, including those involving PIEZO2 and AMPA receptors. Previously, we demonstrated that loss of *UBE3A* expression leads to elevated cofilin levels, reduced F-actin, and impaired PIEZO2 mechanocurrents (Romero et al., 2023). Several studies have shown that force-from-filament (FFF) mechanisms modulate the function of PIEZO channels, and disruption of cytoskeletal components—such as actin, tubulin, or β-catenin—leads to impaired mechanotransduction (Eijkelkamp et al., 2013; Romero et al., 2020; Verkest et al., 2022). This functional interplay may offer a compensatory advantage: stabilizing the cytoskeleton could help mitigate the consequences of pathogenic *PIEZO2* LOF phenotypes. Indeed, one of our key findings is that the cofilin inhibitor SZ-3 restores mechanocurrents (including those of PIEZO2) and mechanoexcitability in DRG neurons from an AS mouse model by increasing their F-actin and Young’s modulus. Notably, by optogenetically severing F-actin with *Z*-lock cofilin, we could identify the role of intact filaments in mechanotransduction. This disruption occurred within minutes, faster than membrane protein trafficking, which generally occurs over larger timescales. Our data support the notion that actin dynamics acutely fine-tune the function of mechanosensitive ion channels. This, however, does not contradict the force-from-lipids (FFL) principle but instead suggests that FFF and FFL mechanisms act together to modulate mechanosensitivity, as previously proposed (Goodman et al., 2023).

Our earlier work showed that prolonged disruption of F-actin with latrunculin A—mimicking the effects of continuously elevated cofilin levels in the DRG neurons of *Ube3a*-deficient mice—leads to reduced membrane expression of PIEZO2 (Romero et al., 2023). Therefore, our results suggest that in AS, actin filament disorganization may impair both the long-term trafficking of PIEZO2 to the plasma membrane and the rapid, second-scale neuronal mechanics required for precise mechanosensation. A previous work showed that hippocampal neurons have a reduction in F-actin in AS (Baudry et al., 2012). Actin, the primary cytoskeletal element in mature synapses, is essential for synaptogenesis, maintenance, and plasticity (Shaw and Koleske, 2021). AMPA receptor density is reduced in hippocampal excitatory synapses, a defect that may contribute to the cognitive impairment observed in AS (Greer et al., 2010). Our findings confirm impaired AMPA receptor function in AS and identify cofilin inhibition as a strategy to improve channel function.

Motor and cognitive deficits are among the most common and debilitating aspects of AS (Margolis et al., 2015). Our finding that SZ-3 ameliorates these phenotypes in an AS mouse model highlights potential alternative therapeutic targets beyond UBE3A. We have recently shown that SZ-3 possesses favorable pharmacological and pharmacokinetic profiles for treating neurodegenerative disorders (Alsegiani and Shah, 2023). Given the widespread expression of cofilin across nearly all cell types, systemic administration may be suboptimal. Future studies should focus on developing targeted delivery strategies to improve the actin cytoskeleton solely in mechanoreceptor and hippocampal neurons. Although SZ-3 enhanced PIEZO2 channel and AMPA receptor function, as well as improved performance in the rotarod and T-maze tests, our study does not exclude the possibility that its behavioral effects may also involve mechanisms beyond the enhancement of neuronal ion channel function. Nevertheless, our work provides proof of concept that inhibiting cofilin is a potential strategy for improving motor coordination and spatial learning in AS.
